# CSF Levels of Baseline VCAM-1 and ICAM-1 Are Associated with Tau Pathology in Patients Demonstrating Cognitive Impairment

**DOI:** 10.3390/neurolint18050084

**Published:** 2026-04-29

**Authors:** Manal Aljuhani, Azhaar Ashraf, Abdullah Alqarni, Mohammed S. Alshuhri, Essam Mohammed Alkhybari, Amani Alharbi, Alanoud Almudayni, Fatmah Jamal Alablani, Ahmad A. Alhulail

**Affiliations:** 1 Department of Radiology and Medical Imaging, College of Applied Medical Sciences, Prince Sattam Bin Abdulaziz University, Al-Kharj 16278, Saudi Arabia; aa.alqarni@psau.edu.sa (A.A.); m.alshuhri@psau.edu.sa (M.S.A.); e.alkhybari@psau.edu.sa (E.M.A.); ama.alharbi@psau.edu.sa (A.A.); a.almudayni@psau.edu.sa (A.A.); f.alablani@psau.edu.sa (F.J.A.); a.alhulail@psau.edu.sa (A.A.A.); 2 Radiological Sciences and Technology Research Unit, College of Applied Medical Sciences, Prince Sattam Bin Abdulaziz University, Al-Kharj 16278, Saudi Arabia; 3 Division of Neurology, Department of Brain Sciences, Faculty of Medicine, Imperial College London, London W12 0NN, UK; azhaar.ashraf@imperial.ac.uk

**Keywords:** CSF, AD, tau, Aβ, VCAM-1, ICAM-1

## Abstract

Background: Vascular dysfunction and neurovascular inflammation are increasingly recognized as contributors to Alzheimer’s disease (AD) pathophysiology, particularly through interactions with tau-related neurodegeneration. Endothelial adhesion molecules, including vascular cell adhesion molecule-1 (VCAM-1) and intercellular adhesion molecule-1 (ICAM-1), play key roles in blood–brain barrier regulation and immune-vascular crosstalk, yet their relevance to long-term disease progression and established AD biomarkers remains incompletely understood. Methods: Using data from the Alzheimer’s Disease Neuroimaging Initiative (ADNI), we examined associations between baseline cerebrospinal fluid (CSF) levels of VCAM-1 and ICAM-1 and clinical progression, CSF biomarkers, neuroimaging measures, and cognitive outcomes over up to 10 years of follow-up. This study included 294 participants (87 cognitively normal, 129 with mild cognitive impairment, and 78 with AD). Multivariable logistic regression was used to assess associations with diagnostic progression, and linear regression models examined relationships with baseline and longitudinal measures of tau, amyloid-β, hippocampal volume, Fluorodeoxyglucose-Positron Emission Tomography (FDG-PET) metabolism, and cognition. Models were adjusted for age, sex, apolipoprotein E epsilon 4 (APOE ε4) status, baseline diagnosis, and baseline CSF amyloid-β, with false discovery rate correction applied for multiple comparisons. Results: Baseline CSF VCAM-1 and ICAM-1 levels did not differ across diagnostic groups. However, higher baseline levels of both markers were nominally associated with increased odds of disease progression. Notably, ICAM-1 showed a strong and robust association with baseline CSF phosphorylated tau, which remained significant after multiple-comparison correction. VCAM-1 was also associated with tau pathology, though this did not survive correction. Neither marker was associated with baseline or longitudinal changes in hippocampal volume, FDG-PET metabolism, or cognitive performance. Conclusion: CSF VCAM-1 and ICAM-1 appear to reflect neurovascular inflammatory processes linked to tau pathology rather than markers of clinical stage or longitudinal neurodegeneration. These findings support a role for endothelial activation in AD pathophysiology and highlight vascular–immune mechanisms as potential contributors to tau-related disease vulnerability.

## 1. Introduction

Alzheimer’s disease (AD) is a progressive neurodegenerative disorder characterized by the accumulation of amyloid-β (Aβ) plaques and neurofibrillary tangles composed of hyperphosphorylated tau, leading to synaptic dysfunction, neuronal loss, and progressive cognitive decline [1]. Although Aβ and tau pathology remain central to current disease models, increasing evidence indicates that vascular dysfunction and immune dysregulation play critical roles in the initiation and progression of AD, including during its prodromal phase, mild cognitive impairment (MCI) [2,3]. Disruption of the neurovascular unit, endothelial activation, and altered immune–vascular crosstalk are now recognized as early contributors to neurodegeneration and tau-related pathology, highlighting neurovascular inflammation as an integral component of AD pathophysiology.

Among markers of vascular inflammation, vascular cell adhesion molecule-1 (VCAM-1) and intercellular adhesion molecule-1 (ICAM-1) have received particular attention due to their roles in leukocyte adhesion, endothelial activation, and regulation of blood–brain barrier (BBB) permeability [4,5]. These adhesion molecules are expressed by activated endothelial cells and facilitate immune cell trafficking into the central nervous system, processes that may amplify neuroinflammation and downstream neuronal injury. Unlike other endothelial markers or components of the neurovascular unit, such as E-selectin or vascular endothelial growth factor (VEGF), which primarily reflect transient endothelial activation or angiogenic signalling, VCAM-1 and ICAM-1 directly mediate immune cell adhesion and trans-endothelial migration across the BBB, positioning them at a critical mechanistic intersection between vascular dysfunction, sustained neuroinflammation, and neurodegeneration. Their relevance to AD is further supported by experimental evidence linking endothelial activation to microglial recruitment, tau propagation, and synaptic dysfunction [4,5,6].

The identification of biomarkers reflecting these non-amyloid, non-tau mechanisms is increasingly important, particularly for detecting AD at preclinical or early symptomatic stages when disease-modifying interventions may be most effective [7,8]. Vascular and inflammatory biomarkers offer complementary biological information beyond classical AD markers, providing insight into endothelial dysfunction, immune activation, and neurovascular integrity. Integrating such markers into existing biomarker frameworks may improve disease stratification, prognostication, and mechanistic understanding, thereby supporting a shift toward earlier and more personalised therapeutic strategies [9]. Recent population-based evidence further supports this concept, demonstrating that higher circulating levels of ICAM-1 and VCAM-1 are associated with poorer cognitive performance in older adults without dementia, suggesting that vascular inflammation contributes to cognitive vulnerability even before overt neurodegenerative disease becomes clinically apparent [10].

Several studies have reported elevated levels of VCAM-1 and ICAM-1 in plasma and cerebrospinal fluid (CSF) in individuals with AD and MCI compared with cognitively unimpaired controls [11,12,13]. Importantly, longitudinal evidence from the Swedish BioFINDER cohort, led by Hansson and colleagues, demonstrated that CSF levels of ICAM-1 and VCAM-1 increase across the AD continuum, including preclinical, prodromal, and dementia stages, with changes detectable up to several years before symptom onset [14]. In that study, higher CSF concentrations of ICAM-1 and VCAM-1 were associated with elevated total tau and markers of neurodegeneration, with stronger associations observed in Aβ-positive individuals. Moreover, these adhesion molecules were linked to cortical thinning in regions such as the precuneus and superior parietal cortex, suggesting a relationship between endothelial activation, tau-related neurodegeneration, and region-specific structural brain changes.

In contrast, not all longitudinal studies have reported consistent associations between inflammatory or endothelial markers and cognitive decline. For example, van Setten et al. recently reported that plasma inflammatory cytokines (TNF-α and IL-1β) and endothelial markers, including E-selectin, ICAM-1, and VCAM-1, showed neither cross-sectional nor longitudinal associations with cognitive decline over an 18-month follow-up period in patients with AD [15]. Differences between such findings and those from BioFINDER and other cohorts may reflect variability in study design, follow-up duration, disease stage at inclusion, biological compartment assessed (plasma versus CSF), and outcome measures. Notably, shorter follow-up intervals, limited group stratification by amyloid or tau status, and reliance on global cognitive outcomes rather than regional neuroimaging markers may reduce sensitivity to early or subtle disease-related changes.

While VCAM-1 and ICAM-1 are not specific to AD and are elevated in other neuroinflammatory and vascular conditions, their relevance to AD lies in their consistent association with endothelial dysfunction, BBB disruption, and tau-related neurodegeneration rather than disease specificity per se [14,16,17]. Increasing evidence suggests that these molecules reflect a vascular-immune axis that interacts with core AD pathology, particularly in Aβ-positive individuals, positioning them as candidate biomarkers of disease mechanisms rather than diagnostic markers alone [14,16,17,18,19].

The present study builds on our previous work examining CSF concentrations of TNF-α and its receptors across the AD continuum [20] by extending the focus to endothelial adhesion molecules. Using the same AD Neuroimaging Initiative (ADNI) cohort, we aimed to investigate whether baseline CSF levels of VCAM-1 and ICAM-1 are associated with long-term clinical progression over a 10-year follow-up period. We further examined relationships between these adhesion molecules and established AD biomarkers, structural neuroimaging measures, and cognitive outcomes. By leveraging extended longitudinal follow-up and multimodal phenotyping, this study seeks to clarify the potential role of VCAM-1 and ICAM-1 as markers of neurovascular inflammation relevant to disease progression while contextualizing our findings within the broader and sometimes contrasting literature.

## 2. Methods

### 2.1. ADNI Study

The dataset utilized in this study was sourced from the ADNI database (http://adni.loni.usc.edu/, accessed on 20 April 2026) in September 2024, including participants from the ADNI-1 and ADNI-GO cohorts. All participants provided informed consent prior to data collection and publication. Genotyping for the apolipoprotein E (APOE) ε4 allele was conducted for all participants. The ADNI protocol incorporates comprehensive clinical, neuropsychological, neuroimaging, and CSF biomarker assessments to longitudinally track disease progression. Ethical approval for the ADNI study was granted by the institutional review boards of all participating sites, and written informed consent was obtained from each participant. All procedures involving human participants complied with the ethical standards of the relevant institutional and national research committees, as well as with the 1964 Declaration of Helsinki and its subsequent amendments or equivalent ethical guidelines. Additional information, including details about the data used in this study, can be accessed at the ADNI website (http://adni.loni.usc.edu/, accessed on 20 April 2026).

### 2.2. CSF Measurements for VCAM1 and ICAM1

The CSF analyses were performed by two experienced research scientists with extensive experience with the multiplex assay techniques and blinded for diagnosis and other subject related information. CSF standards were interspersed, and the levels of CSF inflammatory proteins were adjusted using CSF standard values across the plates. For the analytes of interest, the 96 well ADNI CSF samples were first randomly interspersed amongst 96-well plates and then measured for biomarker levels in the same 2-day time block to avoid repeated freeze–thaw cycles. CSF standards were included on each plate, and all samples were run in duplicate. The CSF protein values were normalized across plates to values of the CSF standards, with an inter-plate CV used to calculate intermediate precision for each analyte.

CSF measurements for Aβ_42_, total tau, and p-tau181 have been described in detail previously [21,22,23].

### 2.3. Inclusion/Exclusion Criteria

Individuals enrolled in the ADNI-1 cohort were between 55 and 90 years of age and required a study partner who could independently provide an accurate assessment of the participant’s daily functioning. All participants were fluent in either English or Spanish. Eligibility criteria included a Hachinski Ischemic Score of ≤4, a Geriatric Depression Scale score below 6, and adequate visual and auditory capabilities to complete neuropsychological assessments. Participants were also required to have at least a sixth-grade education or equivalent occupational experience and could not be simultaneously enrolled in other clinical trials or research studies.

Exclusion criteria comprised the use of psychoactive or centrally acting medications, such as narcotic analgesics, neuroleptics, anticholinergic agents, antiparkinsonian drugs, investigational agents, benzodiazepines, and commonly prescribed central nervous system–active antihypertensives or antidepressants, within four weeks prior to screening. Participants with a history of neurological disorders other than AD, significant brain lesions, or traumatic brain injury were also excluded from the study.

Cognitively normal (CN) participants were required to show no measurable deficits across cognitive domains, maintain independent activities of daily living, and have Mini-Mental State Examination (MMSE) scores between 24 and 30. In addition, CN participants had to have a Clinical Dementia Rating (CDR) of 0, show no symptoms of depression, and demonstrate no evidence of mild cognitive impairment (MCI) or dementia. CN subjects were age-matched to participants in the MCI and AD groups to ensure demographic comparability.

Participants classified as having MCI were required to have MMSE scores ranging from 24 to 30, self- or informant-reported subjective memory complaints, and objective memory impairment based on education-adjusted scores on the Wechsler Memory Scale Logical Memory II below established cutoffs. They were also required to have a CDR of 0.5, indicating very mild cognitive impairment, but preserved functioning in other cognitive domains and intact activities of daily living. Importantly, participants in this group did not meet diagnostic criteria for dementia.

The AD group included individuals with MMSE scores between 20 and 26, CDRs of 0.5 or 1.0, and a clinical diagnosis consistent with the National Institute of Neurological and Communicative Disorders and Stroke–AD and Related Disorders Association (NINCDS–ADRDA) criteria for probable AD.

### 2.4. Diagnostic and Progression Assessment Procedures

Monitoring disease progression is a key objective within the ADNI framework. For each study visit, site investigators conduct a comprehensive review of the participant’s clinical, cognitive, and neuroimaging data to complete a diagnostic summary. When a change in diagnosis occurs—such as a transition from CN to MCI, or from MCI to AD–the corresponding neuropsychological evaluations for that visit are independently reviewed by an onsite clinical monitor to verify diagnostic accuracy.

If discrepancies arise, the site’s principal investigator may discuss the case with the clinical monitor, who retains the authority to question or recommend reversal of a diagnostic decision if it appears inconsistent with the collected data. The ADNI clinical co-investigator subsequently reviews all relevant documentation and works with the clinical monitor to resolve any inconsistencies in scoring or diagnostic interpretation. Following this step, the ADNI conversion committee conducts a centralized review of all diagnostic summaries. In accordance with the NINCDS-ADRDA procedures manual (see http://adni.loni.usc.edu/, accessed on 20 April 2026), the committee reaches a consensus determination regarding each participant’s conversion status.

Although neuropathological confirmation remains the definitive standard for diagnosing AD, extensive validation within the ADNI cohort has demonstrated high sensitivity and specificity for diagnostic classifications based on longitudinal neuroimaging, cognitive testing, and clinical assessments. These multimodal approaches provide reliable markers of disease progression and are widely regarded as sufficient for tracking the clinical transition toward AD in research settings [24,25,26].

### 2.5. Structural MRI Volumes

All participants underwent magnetic resonance imaging (MRI) on 1.5 Tesla scanners using a three-dimensional sagittal volumetric magnetization-prepared rapid gradient-echo (MP-RAGE) sequence. The imaging parameters were as follows: repetition time (TR) = 9 ms, echo time (TE) = 4 ms, and flip angle = 8°. Images were acquired with a 256 × 256 × 170 matrix across the x-, y-, and z-axes, resulting in a voxel resolution of 0.94 × 0.94 × 1.2 mm^3^. MRI scans were obtained at baseline, followed by sessions at 6 months, 12 months, and annually thereafter for up to 6 years.

Quantification of hippocampal volume was performed using the FreeSurfer image analysis suite (version 4.1.0). Volumes were calculated as the product of the total number of hippocampal voxels and the individual voxel volume, following the standardized ADNI image processing pipeline as previously reported [27]. This automated segmentation approach ensured consistency and reproducibility across time points and imaging sites.

Hippocampal volumes obtained from FreeSurfer segmentations were visually inspected for segmentation accuracy and anatomical consistency across all time points. Any scans exhibiting significant motion artifacts, poor registration, or segmentation errors were excluded from further analysis. To account for individual differences in head size, hippocampal volumes were normalized to intracranial volume (ICV), which was also derived from FreeSurfer outputs using the automated whole-brain segmentation routine.

Longitudinal changes in hippocampal volume were quantified by calculating annualized atrophy rates between consecutive MRI sessions. These rates were expressed as a percentage of baseline hippocampal volume to provide a standardized measure of structural decline over time. Participants with at least two high-quality scans were included in the longitudinal analyses. All volumetric data underwent quality assurance through centralized ADNI image processing protocols, which include automated outlier detection and manual verification by trained analysts to ensure reliability and reproducibility across imaging sites.

### 2.6. [18F] Fluorodeoxyglucose ([18F]FDG-PET)

[18F]FDG-PET positron emission tomography ([18F]FDG-PET) imaging was conducted across multiple sites using scanners with varying spatial resolutions [28]. PET scans were obtained at 6 months, and subsequently at 1-, 1.5-, and 2-years following baseline assessment. Each acquisition consisted of six consecutive 5-min frames, beginning 30 min after intravenous injection of 5 mCi of [18F]FDG.

All PET datasets were processed according to the standardized ADNI imaging protocol. Individual frames were first co-registered, averaged, and reoriented along the anterior–posterior commissure (AC-PC) line, after which the resulting volumes were resampled to an isotropic voxel size of 1.5 mm^3^. Spatial normalization was performed using the Montreal Neurological Institute (MNI) template space to allow for inter-subject comparability.

For each participant, regional glucose metabolism was quantified by calculating mean hippocampal FDG uptake, normalized to the pons as a reference region. This normalization approach minimizes inter-scanner variability and allows for robust comparison of relative metabolic activity across time points and diagnostic groups.

### 2.7. Neuropsychological Assessments

Cognitive performance was evaluated using two well-established neuropsychological measures sensitive to disease progression in AD: the Rey Auditory Verbal Learning Test (RAVLT) and the AD Assessment Scale–Cognitive Subscale, 13-item version (ADAS-Cog13).

The RAVLT was administered to assess episodic verbal learning and memory. During this task, participants were asked to learn a list of 15 unrelated words presented over five consecutive learning trials. After the learning phase, a short-delay recall trial was conducted, followed by exposure to an interference (distractor) word list. Subsequently, a 30-min delayed recall trial and a recognition trial (yes/no format) were completed to evaluate retention and retrieval performance (ADNI Procedures Manual). This test is particularly sensitive to early memory impairment and is frequently used to monitor cognitive changes associated with MCI and AD progression.

The ADAS-Cog13 is an expanded 13-item version of the original ADAS-Cog, designed to measure multiple domains of cognitive function, including learning and memory, language comprehension and production, praxis (constructional and ideational), orientation, and attention. The scale also includes number cancellation and delayed free recall tasks to enhance sensitivity to subtle cognitive decline. The word recall component was administered at the beginning of the test, while the word recognition task appeared near the end, with intervening cognitive tasks placed between them to reduce potential interference between the two memory measures.

Following the completion of the objective cognitive tests, a subjective clinical evaluation was conducted by the examiner to assess the participant’s language laterality, short-term memory capacity, and ability to comprehend and retain task instructions. These evaluations provided additional context for interpreting the participant’s cognitive profile. Detailed testing and scoring procedures followed the standardized ADNI protocol, as described in the ADNI General Procedures Manual (more details available on https://adni.loni.usc.edu/wp-content/uploads/2024/02/ADNI_General_Procedures_Manual.pdf, accessed on 20 April 2026).

### 2.8. Statistical Analysis

As this study represents a secondary analysis of data obtained from ADNI, no a priori sample size calculation was performed. The sample size was determined by the availability of participants with complete CSF ICAM-1 and VCAM-1 measurements and relevant clinical, imaging, and biomarker data.

Group differences in CSF ICAM-1 and VCAM-1 levels, clinical, imaging, and biomarker data across CN, MCI, and AD participants were examined using one-way analysis of variance (ANOVA), followed by Tukey’s post hoc test when significant effects were observed. To account for potential heterogeneity of variances, Brown-Forsythe and Welch ANOVA tests were used.

Statistical analyses were conducted to investigate the associations between CSF ICAM-1 and VCAM-1 levels and clinical, cognitive, and neuroimaging markers of AD progression. Disease progression was defined as a binary outcome and examined using multiple logistic regression models, with CSF ICAM-1 and VCAM-1 entered as independent predictors in separate models. All logistic regression models were adjusted a priori for relevant covariates, including age, sex, APOE ε4 carrier status, baseline clinical diagnosis, and baseline CSF Aβ levels. To obtain parsimonious models, a backward elimination approach was applied, whereby covariates with the highest non-significant *p* values were sequentially removed while retaining the primary predictors of interest. Final models are reported as odds ratios (ORs) with 95% confidence intervals.

To evaluate associations between CSF ICAM-1 and VCAM-1 levels and baseline as well as longitudinal measures, multiple linear regression analyses were performed. Outcomes of interest included FDG-PET SUVR, hippocampal volume, cognitive performance as assessed by ADAS-Cog 13 and RAVLT, as well as CSF biomarkers of Aβ and tau. For longitudinal analyses, annual change in each outcome measure was modelled as the dependent variable. Linear regression models were adjusted for the same set of covariates used in the logistic regression analyses (age, sex, APOE ε4 status, baseline diagnosis, and baseline CSF Aβ levels).

A backward elimination procedure was applied to reduce model complexity, with variables exhibiting the highest non-significant *p* values removed in a stepwise manner to achieve parsimonious final models. Regression coefficients (β) with corresponding 95% confidence intervals were reported for linear models. Model assumptions were evaluated through inspection of residuals, and multicollinearity was assessed using variance inflation factors. To account for multiple testing across regression models, false discovery rate (FDR) correction was applied using the two-stage Benjamini, Krieger, and Yekutieli procedure, with a q < 0.05 considered statistically significant. All statistical analyses were performed using GraphPad Prism 10.6.0.

## 3. Results

A total of 294 participants were included in the analysis, comprising 87 CN individuals, 129 participants with MCI, and 78 patients with AD. Demographic characteristics were comparable across diagnostic groups (Table 1). Mean age did not differ significantly between groups (*p* = 0.382), and the proportion of female participants was similar across cohorts (*p* = 0.419).

In contrast, marked differences were observed in genetic, biomarker, neuroimaging, and cognitive measures. The frequency of APOE ε4 carriers increased progressively across the clinical spectrum, from CN individuals (25%) to those with MCI (53%) and AD (71%), with a highly significant group effect (*p* = 2.5 × 10^−8^).

### 3.1. AD Biomarkers

CSF biomarkers showed patterns consistent with AD pathology. CSF Aβ42 levels were highest in cognitively normal participants and decreased stepwise in the MCI and AD groups (*p* = 3.1 × 10^−15^). Conversely, CSF total tau and phosphorylated tau concentrations increased across diagnostic categories, with the lowest levels observed in CN individuals and the highest in AD patients (total tau: *p* = 5.7 × 10^−9^; phosphorylated tau: *p* = 2.6 × 10^−10^).

Neuroimaging measures also differed significantly between groups. FDG-PET standardized uptake value ratios declined progressively from CN participants to MCI and AD patients (*p* < 1 × 10^−15^), indicating increasing cerebral hypometabolism. Similarly, hippocampal volume was significantly reduced across the disease continuum, with the greatest atrophy observed in the AD group (*p* < 1 × 10^−15^).

Cognitive performance showed robust group differences across all assessments. Episodic memory performance, measured by the RAVLT, decreased substantially from CN individuals to MCI and AD patients (*p* < 1 × 10^−15^). Global cognitive function, as assessed by the MMSE, also declined across groups (*p* < 1 × 10^−15^). Disease severity measures demonstrated a corresponding pattern, with progressively higher ADAS-Cog 13 and CDR-SB scores observed from CN participants to those with MCI and AD (both *p* < 1 × 10^−15^).

### 3.2. Baseline Levels of CSF ICAM-1 and VCAM-1

**Figure 1:**
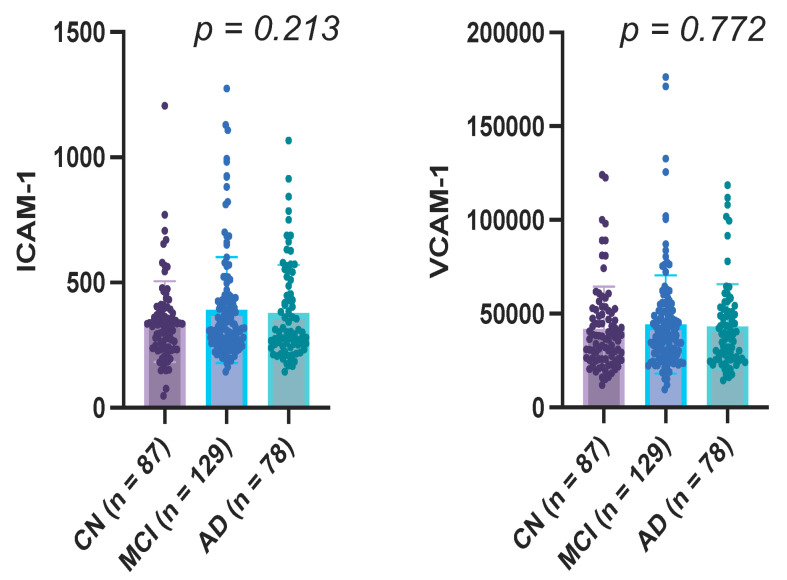
One-way Analysis of variance showing no significant differences for ICAM-1 and VCAM-1 between cognitively normal (CN), mild cognitive impairment (MCI), and Alzheimer’s disease (AD).

No significant differences in mean CSF ICAM-1 (*p* = 0.213, q = 0.583) or VCAM-1 (*p* = 0.772, q = 0.817) levels were observed between CN, MCI, and AD (Figure 1).

The median follow-up of CN and MCI participants who remained stable (n = 107) was 3 years (range 1–10 years) while those in the disease progression group (n = 97) was 4 years (range 1–10 years). In the latter group, the median time to disease progression was two years (range 1–10 years).

In multivariable logistic regression analyses adjusted for age, sex, APOE ε4 carrier status, baseline diagnosis, and baseline CSF amyloid levels, higher CSF ICAM-1 levels were positively associated with increased odds of disease progression (odds ratio = 0.00232; 95% CI: 0.00036 to 0.00455; *p* = 0.0192). CSF VCAM-1 was positively associated with progression risk (OR = 2.18 × 10^−5^; 95% CI: 4.61 × 10^−6^ to 4.14 × 10^−5^; *p* = 0.012). These associations did not remain statistically significant after correction for multiple comparisons using the two-stage Benjamini, Krieger, and Yekutieli false discovery rate procedure (q = 0.131 [ICAM-1]; q = 0.108 [VCAM-1]).

### 3.3. Association of Baseline Levels of CSF ICAM-1 with Baseline Aβ, Tau, Neuroimaging and Cognitive Measures

Using covariate-adjusted multiple linear regression analyses, baseline CSF ICAM-1 levels were not significantly associated with baseline levels of FDG-PET SUVR (F [1, 143] = 2.12, *p* = 0.1477, q = 0.504), hippocampal volume (F [1, 200] = 3.643, *p* = 0.0578, q = 0.263), ADAS-Cog 13 (F [1, 254] = 2.591, *p* = 0.1087, q = 0.424), RAVLT (F [1, 253] = 0.4634, *p* = 0.4967, q = 0.753), and CSF Aβ (F [1, 255] = 0.57, *p* = 0.452, q = 0.753). Notably, higher CSF ICAM-1 levels were significantly associated with higher baseline p-tau concentrations (F [1, 250] = 31.18, *p* = 6.11 × 10^−8^, q = 8.33 × 10^−7^), independent of demographic and clinical covariates.

### 3.4. Association of Baseline Levels of CSF ICAM-1 with Annual Changes in Aβ, Tau, Neuroimaging and Cognitive Measures

CSF ICAM-1 levels were not significantly associated with annual changes in cerebral glucose metabolism as measured by FDG-PET SUVR (F [1, 74] = 0.005, *p* = 0.943, q = 0.919). Similarly, no significant associations were observed between CSF ICAM-1 and annual changes in hippocampal volume (F [1, 131] = 0.69, *p* = 0.408, q = 0.742). No significant relationships were detected between CSF ICAM-1 levels and longitudinal cognitive change, as assessed by ADAS-Cog 13 (F [1, 169] = 0.29, *p* = 0.593, q = 0.809) or RAVLT (F [1, 169] = 0.77, *p* = 0.382, q = 0.742). In addition, CSF ICAM-1 was not associated with annual changes in CSF Aβ levels (F [1, 156] = 0.33, *p* = 0.569, q = 0.809) or p-tau levels (F [1, 137] = 0.14, *p* = 0.714, q = 0.817).

Summary statistics for longitudinal changes in neuroimaging, cognitive, and biomarker outcomes are provided in Supplementary PDF File S1.

### 3.5. Association of Baseline Levels of CSF VCAM-1 with Baseline Aβ, Tau, Neuroimaging and Cognitive Measures

Using covariate-adjusted multiple linear regression analyses, baseline CSF VCAM-1 levels were not significantly associated with baseline levels of FDG-PET SUVR (F [1, 140] = 0.177, *p* = 0.675, q = 0.817), hippocampal volume (F [1, 198] = 0.938, *p* = 0.334, q = 0.795), ADAS-Cog 13 (F [1, 252] = 0.118, *p* = 0.731, q = 0.817), RAVLT (F [1, 251] = 0.105, *p* = 0.746, q = 0.817), and CSF Aβ (F [1, 255] = 5.087, *p* = 0.025, q = 0.137). Notably, higher CSF VCAM-1 levels were significantly associated with higher baseline p-tau concentrations (F [1, 249] = 63, *p* = 6 × 10^−14^, q = 1.64 × 10^−12^), independent of demographic and clinical covariates.

### 3.6. Association of Baseline Levels of CSF VCAM-1 with Annual Changes in Aβ, Tau, Neuroimaging and Cognitive Measures

CSF VCAM-1 levels were not significantly associated with annual changes in cerebral glucose metabolism, as measured by FDG-PET SUVR (F [1, 93] = 0.78, *p* = 0.378, q = 0.795). Similarly, no significant association was observed between CSF VCAM-1 and annual change in hippocampal volume (F [1, 133] = 1.74, *p* = 0.190, q = 0.577). No significant relationships were detected between CSF VCAM-1 levels and longitudinal cognitive change, as measured by ADAS-Cog 13 (F [1, 169] = 0.52, *p* = 0.473, q = 0.798) or RAVLT (F [1, 169] = 0.06, *p* = 0.809, q = 0.817). In addition, CSF VCAM-1 was not significantly associated with annual changes in CSF Aβ levels (F [1, 156] = 0.15, *p* = 0.699, q = 0.817) or p-tau levels (F [1, 136] = 0.07, *p* = 0.797, q = 0.817).

## 4. Discussion

In this study, we investigated the relationship between the endothelial adhesion molecules ICAM-1 and VCAM-1, as markers of neurovascular inflammation, across the clinical spectrum using data from the ADNI cohort. Several key findings emerged. Baseline CSF ICAM-1 and VCAM-1 levels did not differ significantly across diagnostic groups. However, both markers were associated with tau pathology at baseline, with a particularly strong and robust association observed for ICAM-1 that remained significant after correction for multiple comparisons. In contrast, neither ICAM-1 nor VCAM-1 was associated with longitudinal changes in neuroimaging measures, cognitive performance, or CSF biomarkers. These findings suggest that endothelial adhesion molecules are linked to existing pathological processes, particularly tau pathology, rather than serving as markers of subsequent disease progression.

One of the most robust findings was the strong association between CSF ICAM-1 and baseline CSF p-tau, which remained significant after false discovery rate correction. This result aligns with accumulating evidence that endothelial activation and BBB dysfunction are closely linked to tau pathology and neurodegeneration rather than amyloid burden alone [14,15,16,17]. ICAM-1 is a key mediator of leukocyte adhesion and transendothelial migration, and its upregulation reflects sustained endothelial activation and immune cell recruitment [4,5]. These processes may facilitate microglial activation, cytokine release, and oxidative stress, thereby promoting tau hyperphosphorylation and neuronal injury [29,30]. Given the cross-sectional nature of this association, it remains unclear whether endothelial activation precedes, follows, or develops in parallel with tau accumulation.

CSF VCAM-1 also showed strong associations with tau pathology and a nominal association with CSF Aβ that did not survive multiple-testing correction. While this finding should be interpreted cautiously, it raises important mechanistic questions regarding the role of VCAM-1 in Aβ biology. Experimental studies have implicated the VCAM-1/APOE pathway in microglial recruitment to Aβ plaques and Aβ clearance [6]. Lau and colleagues demonstrated that endothelial VCAM-1 signalling guides microglia toward APOE-associated Aβ plaques, promoting phagocytosis in experimental models [6]. However, the same study reported that in patients with AD, higher CSF levels of soluble VCAM-1 were associated with impaired microglial Aβ chemotaxis, suggesting that elevated soluble VCAM-1 may reflect dysregulated or maladaptive immune-vascular signalling rather than effective clearance [6].

Within this framework, the positive association observed between CSF VCAM-1 and Aβ in the present study may reflect neurovascular injury or BBB dysfunction rather than enhanced amyloid removal. Soluble VCAM-1 in CSF is thought to arise, at least in part, from endothelial shedding in response to vascular stress or inflammation [4,5]. BBB breakdown has been linked to impaired Aβ transport, reduced clearance via perivascular drainage pathways, and increased vascular amyloid deposition [4,5,29,30]. Thus, elevated CSF VCAM-1 may serve as a marker of endothelial damage and BBB compromise that indirectly contributes to Aβ accumulation. This interpretation is consistent with emerging models positioning vascular inflammation as a facilitator of AD pathology rather than a compensatory protective response.

In contrast to the cross-sectional findings, neither ICAM-1 nor VCAM-1 was associated with longitudinal changes in hippocampal volume, FDG-PET metabolism, cognitive performance, or CSF biomarkers. This distinction is important. It suggests that while these markers are related to existing pathological burden, they do not appear to capture the rate of subsequent neurodegeneration or cognitive decline within this cohort. In this sense, ICAM-1 and VCAM-1 may be better understood as markers of a neurovascular inflammatory state rather than indicators of disease progression [15,17].

Comparison with prior longitudinal studies provides important context. The Swedish BioFINDER study reported increasing CSF ICAM-1 and VCAM-1 levels across the AD continuum, including preclinical stages, with strong associations with tau pathology, cortical thinning, and neurodegeneration [14]. The present study aligns with prior work in demonstrating associations with tau pathology but does not support a role for these markers in predicting longitudinal change. These differences may reflect cohort composition, duration of follow-up, disease stage at inclusion, or outcome sensitivity. BioFINDER incorporated Aβ-stratified analyses and region-specific cortical thickness measures, which may be more sensitive to early vascular-inflammatory effects than global hippocampal volume or FDG-PET metrics used here.

Although APOE ε4 status was included as a covariate in all analyses, stratified analyses by APOE genotype were not performed. Given strong experimental and clinical evidence implicating APOE in BBB integrity, microglial function, and VCAM-1–mediated signalling [6,16,19], genotype-specific effects remain an important area for future investigation. It is plausible that associations between adhesion molecules and AD pathology are amplified or qualitatively different in APOE ε4 carriers, as suggested by prior studies [14,15,16,17].

Several limitations should be acknowledged. ICAM-1 and VCAM-1 were measured only at baseline, precluding evaluation of temporal changes in neurovascular inflammation. Serial measurements of endothelial markers may help better characterise their temporal relationship with AD-related processes, clarifying whether endothelial activation precedes, parallels, or follows tau accumulation. Variability in follow-up duration and progression rates across participants may reduce sensitivity to detect subtle longitudinal effects. The ADNI cohort is highly selected, with limited vascular comorbidity and underrepresentation of racially and socioeconomically diverse populations, which may limit generalizability and underestimate vascular contributions to AD pathology [24,25,26].

Despite these limitations, the present findings provide evidence that CSF ICAM-1 and VCAM-1 are associated with tau pathology in AD. These results support the concept that neurovascular inflammatory processes are linked to core features of AD biology, even in the absence of clear longitudinal effects. Future studies incorporating repeated measurements of endothelial markers, more diverse populations, and multimodal assessments of blood–brain barrier function and neuroinflammation will be important to further clarify the role of vascular-immune mechanisms in AD.

## Figures and Tables

**Table 1:** Demographic, genetic, biomarker, neuroimaging, and cognitive characteristics of the ADNI population.

	Cognitively Normal	Mild Cognitive Impairment	Alzheimer’s Disease	*p*
*n*	87	129	78	
Age, years (mean ± SD)	75.7 ± 5.45	74.3 ± 7.72	74.9 ± 8.19	0.382
* Sex (female, %)	39 (45%)	48 (37%)	35 (45%)	0.419
* APOEε4 carriers (n, %)	22 (25%)	69 (53%)	55 (71%)	2.5 × 10^−8^
^†^ CSF Aβ42 (pg/mL, median [IQR])	1288 (804–1582)	679 (562–1189)	534 (437–729)	<1 × 10^−15^
^†^ CSF total tau (pg/mL, median [IQR])	217 (180–267)	289 (231–365)	341 (269–419)	<1 × 10^−15^
^†^ CSF phosphorylated tau (pg/mL, median [IQR])	20 (16–25)	28 (21–37)	34 (26–44)	<1 × 10^−15^
FDG-PET SUVR	1.28 ± 0.14	1.16 ± 0.14	1.06 ± 0.11	<1 × 10^−15^
Hippocampal volume (mm^3^)	7253 ± 768	6261 ± 1142	5830 ± 1101	<1 × 10^−15^
RAVLT score	42.7 ± 8.9	30.1 ± 8.8	23.1 ± 7.6	<1 × 10^−15^
ADAS-Cog 13 score	9.9 ± 4.3	18.7 ± 6.2	28.8 ± 7.9	<1 × 10^−15^
MMSE	29 ± 1.1	26.9 ± 1.8	23.6 ± 1.9	<1 × 10^−15^
^†^ CDR SB (median [IQR])	0.0 (0.0–0.0)	1.5 (1.0–2.0)	4.0 (3.5–5.0)	<1 × 10^−15^

One-way ANOVA was used for most of the measures except for * Chi-squared test used for computing *p* values for sex and APOEε4 carriers. ^†^ Brown-Forsythe and Welch ANOVA test was used for CSF levels of Aβ, total tau, and phosphorylated tau, to account for potential heterogeneity of variances.

## Data Availability

Data used in preparation of this article were obtained from the ADNI database (http://adni.loni.usc.edu/), accessed on 20 April 2026. As such, ADNI investigators contributed to study design and implementation, and provided data but did not participate in analysis or writing of this report. For a complete listing of ADNI investigators, see https://adni.loni.usc.edu/wp-content/uploads/how_to_apply/ADNI_Acknowledgement_List.pdf, accessed on 20 April 2026. ADNI data are disseminated by the Laboratory for Neuro Imaging at the University of Southern California. All ADNI data are available for public access at adni.loni.usc.edu contingent on adherence to the ADNI Data Use Agreement.

## Supplementary Materials

The following supporting information can be downloaded at: https://www.mdpi.com/article/10.3390/neurolint18050084/s1, PDF File S1: Supplementary ICAM1 and VCAM1 in AD_ADNI.
