# Supplementary material for: CSF Levels of Baseline VCAM-1 and ICAM-1 Are Associated with Tau Pathology in Patients Demonstrating Cognitive Impairment
Source: Neurol Int. 2026 Apr 29;18(5):84. doi: 10.3390/neurolint18050084 (PMC13210058; doi:10.3390/neurolint18050084)
Supplement: Supplementary file 1 [file neurolint-18-00084-s001.zip › neurolint-4201866-supplementary.pdf]

Patient ID	Age	Sex	Diagnosis (baseline)	Conversion status	CSF Aβ stat
4	67.5	Male	LMCI	No	1501
5	73.7	Male	CN	No	547.3
14	78.5	Female	CN	No	1582
16	65.4	Male	CN	No	550.6
23	71.7	Male	CN	No	1647
38	76.8	Male	LMCI	No	874.1
40	73.2	Male	CN	No	1526
42	72.8	Male	LMCI	Yes	1258
45	85.9	Male	LMCI	Yes	383.3
47	84.7	Male	CN	No	1410
51	66.5	Male	LMCI	Yes	352.5
56	69.6	Female	CN	No	1048
57	77.3	Male	LMCI	Yes	597.1
59	70.9	Female	CN	No	1605
61	77	Female	CN	Yes	1288
70	74	Male	CN	No	1427
77	79.7	Male	LMCI	Yes	792.7
89	65.1	Male	CN	No	1414
96	79.6	Male	CN	No	915.3
97	72.8	Female	CN	No	776.6
101	73.6	Male	LMCI	Yes	453.2
107	60.7	Female	LMCI	No	1652
111	75.8	Female	LMCI	Yes	580.2
112	70.6	Male	LMCI	Yes	498.6
120	72	Male	CN	No	1226
123	73.3	Male	CN	Yes	1566
126	77.5	Female	LMCI	Yes	536.8
150	73.9	Male	LMCI	No	817
173	73.2	Male	CN	Yes	991.3
176	74.7	Male	LMCI	No	670.7
177	74.8	Female	CN	No	1367
186	80.4	Female	CN	No	942.8
204	71.1	Female	LMCI	Yes	282.9
210	72.4	Female	CN	No	1385
214	65.1	Male	LMCI	Yes	683.1
223	78	Female	CN	Yes	751.5
232	78	Male	CN	Yes	1028
240	88.3	Male	LMCI	Yes	1338
256	70.1	Male	LMCI	Yes	288.4

259	70.6	Male	CN	Yes	803.6
260	78.6	Female	CN	No	1520
269	67.1	Male	LMCI	Yes	576.8
285	65.6	Male	LMCI	Yes	844.4
292	76.4	Male	LMCI	Yes	941.7
295	84.8	Male	CN	No	888.1
312	82.8	Male	CN	No	838.6
319	70.2	Female	CN	No	1391
327	70	Male	CN	No	1405
331	64.6	Female	LMCI	Yes	588
336	76.1	Male	LMCI	Yes	854.2
344	78.8	Male	LMCI	Yes	556.7
362	70.5	Female	LMCI	Yes	414.1
376	70.5	Male	LMCI	Yes	1148
386	72.3	Female	CN	No	1168
388	71.2	Male	LMCI	Yes	332.6
401	63.9	Male	LMCI	No	1100
403	76.3	Male	CN	No	333.5
410	61.3	Female	LMCI	No	1327
413	76.3	Female	CN	No	1006
429	62.7	Male	LMCI	No	1014
433	85.5	Female	CN	No	831.3
442	74.7	Male	LMCI	Yes	1634
448	85.5	Male	LMCI	Yes	1645
449	66.5	Female	LMCI	No	606
464	83.1	Male	LMCI	No	1203
467	81.1	Female	CN	Yes	500.5
476	75.8	Female	LMCI	Yes	477.6
478	56.1	Male	LMCI	No	1495
479	73.5	Female	CN	No	649.7
481	84.8	Male	LMCI	No	594
501	77.9	Male	LMCI	Yes	568.6
505	79.7	Male	LMCI	No	1600
507	55.2	Female	LMCI	Yes	449.8
511	57.7	Male	LMCI	Yes	627.5
514	80.7	Male	LMCI	Yes	826.8
518	69.4	Male	LMCI	Yes	540.2
519	74.1	Male	CN	No	1263
525	70	Female	CN	No	821.4
534	62.8	Male	CN	No	806.8
544	76.7	Female	LMCI	No	461.6
552	60.1	Female	LMCI	Yes	648.4
555	77	Male	CN	Yes	560.6

559	79.3	Male	CN	No	1245
566	78.8	Male	LMCI	Yes	682.5
567	73.6	Male	LMCI	Yes	368.6
579	65.4	Female	LMCI	No	1429
602	70.7	Male	CN	Yes	1251
604	86.5	Male	LMCI	Yes	1079
605	75.6	Female	CN	Yes	1117
607	78	Female	LMCI	No	507.3
618	74.8	Male	CN	No	1323
621	62.3	Female	LMCI	No	467
638	83.1	Male	LMCI	Yes	269.3
649	86.7	Male	LMCI	Yes	669.3
657	77.7	Female	CN	No	1697
686	72	Male	CN	No	657.6
718	80.4	Female	LMCI	No	803.5
725	80.9	Male	LMCI	Yes	566.7
726	81	Male	CN	No	493.4
729	65.1	Female	LMCI	Yes	957.8
731	71.5	Male	CN	No	744.2
769	62.2	Male	LMCI	Yes	594.2
779	79.5	Male	CN	Yes	742
783	79.2	Female	LMCI	No	1038
792	73.5	Male	LMCI	No	536.8
821	79.2	Male	LMCI	No	1081
839	79.4	Female	LMCI	Yes	730.1
843	70.5	Male	CN	Yes	1298
866	80	Female	CN	No	1271
892	72.8	Female	LMCI	Yes	421.3
896	76.8	Male	CN	Yes	1208
906	65.3	Female	LMCI	Yes	908.9
908	62.9	Female	LMCI	No	1314
915	80.6	Female	LMCI	Yes	509.7
923	83.1	Male	CN	No	1396
930	75	Male	LMCI	Yes	293.7
931	86.1	Female	CN	No	1435
932	75.3	Male	LMCI	No	579.2
941	73.3	Female	LMCI	Yes	755.1
954	69.3	Female	LMCI	Yes	603.1
972	77.9	Male	CN	Yes	696
978	73.1	Male	LMCI	Yes	657.6
994	55.1	Female	LMCI	Yes	699.3
1002	76.3	Female	CN	No	1449
1009	75.8	Male	CN	No	561.3

1033	73.3	Female	LMCI	Yes	907.3
1063	78.4	Female	CN	Yes	685.5
1075	67.1	Male	LMCI	No	670.5
1077	83.3	Male	LMCI	Yes	1516
1126	80.4	Male	LMCI	Yes	566.3
1203	83.3	Male	CN	Yes	1330
1217	67.8	Female	LMCI	Yes	912
1247	72.5	Female	LMCI	Yes	649.1
1250	73.2	Female	CN	No	1643
1260	71.2	Male	LMCI	No	1470
1265	82.2	Female	LMCI	Yes	860.9
1292	76.2	Male	LMCI	Yes	596.6
1293	77.1	Male	LMCI	No	291.1
1309	69.1	Female	LMCI	No	570
1315	83.3	Female	LMCI	Yes	761.5
1321	83.2	Male	LMCI	No	1113
1398	77.6	Female	LMCI	Yes	689.2
1419	77.9	Male	LMCI	No	390.5
1421	74.4	Female	LMCI	No	521.2
33	83.3	Male	LMCI	No	673
41	70.9	Female	LMCI	Yes	414.6
55	75.5	Male	CN	Yes	805.2
66	74.5	Male	CN	No	1477
102	70.3	Female	LMCI	No	539.3
118	80.4	Male	CN	No	1467
190	78.8	Male	LMCI	Yes	674.5
196	78	Female	CN	No	540.6
222	85.9	Male	LMCI	Yes	620.3
293	87.7	Male	LMCI	Yes	702.1
394	84	Female	LMCI	Yes	643.4
441	72.7	Female	CN	No	1614
588	64.6	Male	LMCI	Yes	529.2
598	72.3	Male	LMCI	No	1379
648	71.5	Female	CN	No	1479
778	72.4	Male	CN	Yes	668.2
855	75.6	Male	LMCI	Yes	638.3
878	72.3	Male	LMCI	Yes	526.4
880	84.2	Male	LMCI	No	1243
1035	87.2	Male	CN	No	935.3
1073	65.7	Female	LMCI	Yes	657.9
1120	77.9	Female	LMCI	No	619.8
1200	84.4	Male	CN	Yes	1542
1213	74.8	Female	LMCI	Yes	574

1224	81.1	Male	LMCI	Yes	496.4
1225	76.4	Male	LMCI	No	1275
1295	76.7	Male	LMCI	Yes	614.4
1330	61.7	Male	LMCI	No	907
1352	85.9	Male	LMCI	No	1685
1393	82.5	Male	LMCI	Yes	857.2
8	84.5	Female	CN	Yes	>1700
19	73.1	Female	CN	No	>1700
22	63.2	Male	CN	Yes	>1700
31	77.7	Female	CN	No	>1700
95	71.1	Male	CN	No	>1700
127	70.6	Male	CN	Yes	>1700
158	80.4	Female	LMCI	No	>1700
172	70.6	Female	CN	No	>1700
294	79.2	Male	LMCI	Yes	>1700
326	76.9	Male	LMCI	Yes	>1700
422	61.6	Male	LMCI	No	>1700
443	63	Male	LMCI	No	>1700
446	88.8	Male	LMCI	No	>1700
454	81.8	Female	CN	Yes	>1700
459	72.9	Male	CN	Yes	>1700
498	70.3	Male	CN	No	>1700
516	87.6	Male	CN	No	>1700
610	79	Male	CN	No	>1700
634	82.1	Male	LMCI	No	>1700
680	77.8	Male	CN	Yes	>1700
685	89.6	Female	CN	No	>1700
746	73.8	Female	LMCI	No	>1700
818	74	Female	CN	No	>1700
886	71.3	Female	CN	No	>1700
912	73.9	Female	LMCI	No	>1700
1052	69	Female	LMCI	No	>1700
1140	84.4	Male	LMCI	No	>1700
1227	64.6	Female	LMCI	No	>1700
692	76.7	Female	CN	No	390.9
1276	71.8	Female	CN	No	428.8

	**CSF CAMs (baseline)**		**ADAS-Cog 13**			
APOE status	CSF ICAM1	CSF VCAM	Baseline	1 year	2 year	Annual char
0	221.72	22254.8	21.33	22	NA	0.67
0	474.04	36120.7	14.67	NA	11	-1.835
0	347.05	29204.8	8.33	7.33	11	1.335
1	149.8	25826.9	14.33	12.33	12.33	-1
0	178.48	22678.3	8	8	6	-1
0	302.01	42661.2	16.33	12	NA	-4.33
0	544.03	51511.7	12.33	9	7	-2.665
0	387.84	76250	12	15	18.33	3.165
1	982.31	87118.1	23.33	27.33	NA	4
0	341.95	42532.9	11	5.67	8.67	-1.165
2	259.29	37663.4	17.67	15	16.33	-0.67
0	206.34	15134.3	3	5	NA	2
1	700.32	52983.6	19.67	41	41.67	11
0	338.77	25798.5	10	5.33	NA	-4.67
0	279.43	36373.3	5	13	11	3
0	671.19	53515	14.67	11.33	NA	-3.34
0	925.56	45673.8	27	34.67	35	4
0	276.63	48789.4	3.67	2	5	0.665
0	241.56	27386.4	4	3.33	2.33	-0.835
0	360.59	21808	2.67	3.67	3.33	0.33
2	307.44	12162	11	17	11.67	0.335
0	162.91	24544.3	3	8.67	11.33	4.165
1	338.53	32773.5	25.67	28.33	NA	2.66
1	265.36	61210.5	9	7.67	3.67	-2.665
1	370.77	21600.7	13.33	13	11.67	-0.83
0	1205.05	89166.1	5	15	7.67	1.335
1	284.15	23786.6	14.67	18	24	4.665
0	1108.41	51705.9	22.33	20.33	24.67	1.17
0	341.37	81166.4	14.67	12.33	8.67	-3
1	565.66	55301.7	20.33	21.33	22.33	1
0	335.57	25299.1	6.67	4.67	11	2.165
0	393.11	36549.4	9.67	8	18	4.165
0	323.58	15261.5	18.33	27.67	30	5.835
0	354.5	39434.7	10.67	12.33	12.33	0.83
2	821.26	36561.9	19.33	23.33	24	2.335
1	339.37	44533.1	10.33	23	24.33	7
0	183.82	49600.8	11.67	5.33	8.33	-1.67
0	881.72	54851.4	17.33	16.33	15.67	-0.83
2	280.59	25832.2	26	19	26.67	0.335

0	297.08	21904.2	10.33	6	3.67	-3.33
0	282.72	25797.8	4.33	7.33	9.67	2.67
1	244.93	28881.9	20.67	27.67	29.67	4.5
1	296.89	51144.1	11	12.33	17	3
0	399.36	42463.9	29.67	33	28	-0.835
1	328.41	89080.5	4	5.67	5.67	0.835
1	401.72	59207.5	14.67	15	21.33	3.33
0	478.73	22614	16.33	4	NA	-12.33
0	568.09	40505.7	15	14	12.67	-1.165
2	600.86	36691.7	22	21.33	20.33	-0.835
1	308.27	43408.4	19	16.33	27.33	4.165
2	252.75	51583.6	23.67	23.33	24	0.165
1	811.94	55956.8	20.33	23.67	19	-0.665
0	522.38	27350.2	9	13.67	9.33	0.165
1	353.22	29398.4	13	11	13.67	0.335
2	199.36	41081	21.33	18	23	0.835
1	246.87	41566.6	11.67	14.33	16.33	2.33
0	265.99	47338.2	15	12.67	19	2
1	413.78	24019.3	18.67	17	NA	-1.67
0	162.47	16246.9	4.33	4.33	5.33	0.5
0	202.34	26155.1	16.33	22.33	25.67	4.67
0	469.76	33989.3	7.33	8.67	9	0.835
1	228.65	45796.3	16.67	17.67	NA	1
0	430.75	62239.1	15.67	20	13	-1.335
2	271.11	18978.6	20.67	27	22.67	1
0	384.08	49012.2	13.67	11.33	8	-2.835
0	336.04	32926.7	7	4.67	11.67	2.335
1	291.28	22490.7	NA	35	35.67	0.67
0	231.25	26218.1	7.67	7	14.67	3.5
0	238.42	29977.5	6	3.33	2.67	-1.665
1	260.91	51255.4	13.67	10	14.33	0.33
0	298.71	18535.8	12	18	16.33	2.165
0	259.44	24536.6	16	16.33	8.33	-3.835
0	355.28	23750.4	26.33	30.33	32.67	3.17
0	306.53	42284.8	25.33	22	30.67	2.67
0	144.71	42021.8	15.67	10	12.67	-1.5
2	264.59	35494.1	25	26.67	26.33	0.665
0	298.53	38839.7	6.67	10.67	11.33	2.33
0	254.67	19092.8	5.33	7.33	1.67	-1.83
1	151.31	11834.1	8	7	3.33	-2.335
0	323.24	24473.4	14.33	12	20	2.835
1	228.18	35564.1	23.67	27.67	31	3.665
1	312.71	25771	16	19	13.33	-1.335

1	653.87	46352.3	8	7.67	12.33	2.165
1	570.21	38559	22.67	28	26.67	2
1	660.02	34353.9	32.33	22	34	0.835
1	199.89	48023.1	6	5.67	5.33	-0.335
0	563	43179.6	13	11.67	7.33	-2.835
0	360.58	125508	16.67	15	20.67	2
1	272.03	24995.6	4.67	8.33	8.67	2
1	427.26	42464.4	10.33	16	17.67	3.67
1	232.85	42463.9	8	3.33	6.67	-0.665
1	206.74	18555.3	16.33	19	28.67	6.17
2	315.29	40521.5	28.67	33.33	37.33	4.33
1	523.64	57057	16.67	20	20.67	2
0	356.72	25224.9	15.33	8.67	6	-4.665
0	331.21	52011.7	11	3.33	6	-2.5
0	341.69	30252.8	6	12.33	12.67	3.335
1	252.6	26366.6	18	19	22	2
1	335.72	37410.4	17	NA	18	0.5
1	449	9626.84	14.67	21.67	15.67	0.5
0	411.48	60935.1	5.33	0.33	5.67	0.17
0	179.52	36559	12.33	17.33	19	3.335
1	403.92	61812.9	16	17.33	24.33	4.165
0	280.96	35044.6	19.67	21	18.33	-0.67
0	223.7	22478.3	15	14.33	14	-0.5
1	350.99	76192.5	16	16.33	NA	0.33
0	368.59	56593.5	23	25.67	41	9
0	388.29	52969.3	19.67	18.33	20.33	0.33
0	300.92	30710.9	5.33	7.67	6.67	0.67
1	218.27	22751.4	10.33	11	14.67	2.17
0	215.73	38387.9	6	7.67	8.33	1.165
1	421.74	21628.9	16	20.33	19	1.5
0	212.89	34454.6	8	10.67	18.67	5.335
1	357.81	26521.1	30	34.33	37.67	3.835
0	232.4	43346.2	11.33	17.33	8.67	-1.33
1	425.63	23421.1	25.33	29	25.67	0.17
0	294.28	33032.6	5.33	6.33	14.33	4.5
0	231.78	38357.5	23.67	20.33	27.33	1.83
2	230.59	30192.2	32	46	52	10
1	256	31937.8	21.67	27	33	5.665
1	389.41	124128	9.33	6	7	-1.165
1	1273.99	83791.9	18.67	15.67	21	1.165
1	261.61	48835.9	11.33	18.67	19	3.835
0	260.77	15840	9.33	9.33	5.33	-2
2	707.01	38227.1	8.67	13	15	3.165

1	470.25	50230.2	21.67	22.67	NA	1
1	228.62	30987.7	18.33	13.33	NA	-5
1	275.99	33518.9	15	10.33	10.33	-2.335
0	387.38	55392.7	17.67	14.67	18.67	0.5
0	257.64	33966.2	24	25.33	26	1
0	195.36	26180	10.33	7.67	12.33	1
2	414.14	30419.6	11	20	22.67	5.835
0	344.74	21498	23.33	35.33	35.67	6.17
0	182.22	18213.9	11.33	8.33	7	-2.165
0	278.54	43435.8	16.67	20	16.33	-0.17
1	265.3	24831.5	17	19.33	26	4.5
0	401.87	39813	25	36.67	NA	11.67
2	650.24	36974.4	16	26.67	28.33	6.165
2	266.98	32711.3	14.67	15	18.67	2
0	318.24	45619.4	24.67	27.67	32	3.665
0	327.2	29791.7	27	23	23.33	-1.835
1	370.77	29768.9	29.67	45.67	NA	16
1	286.72	35505.2	14.67	16	16.67	1
1	383.07	23562.5	13	17.33	NA	4.33
0	331.83	38218.4	25.67	27	32.33	3.33
2	233.34	25061.2	28.33	27	30	0.835
0	351.35	31192.6	8.33	10	NA	1.67
0	377.65	52562.1	5.33	3.67	5.67	0.17
1	280.53	15251.4	24.67	35.67	49.33	12.33
0	331.51	40274.6	12.33	13.33	10.33	-1
1	382.39	46558.2	24.67	18	23	-0.835
0	234.14	19299.9	7.67	8.67	8.33	0.33
0	346.11	72290.7	18	22.33	18	0
1	511.26	132785	18	20.33	26	4
0	395.65	42521.2	24	27	32	4
0	336.82	37884.2	2.67	13.67	10.33	3.83
2	309.65	38520.8	29	28	32.33	1.665
0	316.42	64637.6	14.67	12.33	16.67	1
0	197.75	50489.4	19.67	13.33	13	-3.335
1	233.29	31957.3	9.33	15.67	15	2.835
0	400.19	62883.5	19.33	21.33	NA	5
1	262.38	34364.1	22.33	19.67	27.67	2.67
0	258.71	33278.7	17.67	19.33	NA	1.66
1	466.15	122534	10.33	7	8	-1.165
1	451.16	29256.5	24	25	27.33	1.665
0	187.87	46598.4	14.33	14.67	26	5.835
0	338.31	38591.8	14.67	17.67	16.67	1
1	579.57	30010.7	19.33	30.33	49.33	15

0	257.79	34526.1	21	19.67	21	0
0	309.94	58229.9	9	14	NA	5
1	397.17	27348.9	20.33	26	NA	5.67
1	230.88	37968.8	30.33	21.33	21	-4.665
0	439.41	60290.8	13.67	10.67	5	-4.335
0	922.38	75356.2	22	30.33	31.33	4.665
0	579.3	74264.9	7	5	NA	-2
0	265.56	24327.3	4	4.67	NA	0.67
1	305.67	35625.9	13.67	13.33	18.33	2.33
0	431.3	44819.3	1.67	4	3.33	0.83
0	366.35	80920.3	8	8.67	6	-1
0	398.8	56547.8	10.67	15.67	14	1.665
0	310.08	46441.1	11.33	16.67	12.33	0.5
0	247.8	20753.1	12.67	9.67	6.67	-3
0	355.47	102144	16	17.67	23	3.5
0	429.71	47115.3	18.33	18.67	19.33	0.5
0	497.72	44755	10.67	10.67	16.33	2.83
0	295.7	62240.8	7.33	5.67	3.33	-2
0	522.59	176259	20.33	15.33	NA	-5
0	325.36	58422.7	13	7.67	7.67	-2.665
0	309.11	53709.1	15	16.33	20	2.5
1	436.8	30931	7.33	5	4.67	-1.33
0	347.64	98161.9	16.67	15	11.33	-2.67
0	770.15	100009	7	5	3.67	-1.665
0	1129.15	80381.2	21	15.33	19.33	-0.835
0	230.64	44807.1	9.33	11.67	9.67	0.17
0	148.83	60775.7	6.67	9	9.67	1.5
0	266.95	45330.8	11.67	6.67	8	-1.835
0	182.71	30456.6	18	15.33	7.67	-5.165
0	410.32	30393.2	10.33	8.33	4	-3.165
1	442.62	39244.1	19.33	10	12.67	-3.33
1	322.59	45738.2	17.33	12.33	NA	-5
0	276.26	100483	13.33	24	17	1.835
0	332.51	28321	12.67	13.33	16.67	2
1	364.87	20260	11.33	12.67	10.67	-0.33
0	47.72	26497	10	13.67	25.67	7.835

RAVLT				FDG-PET		
Baseline	1 year	2 year	Annual char	Baseline	1 year	Annual char
37	37	NA	0		NA	
37	40	36	-0.5	1.25956	1.2809	0.02134
45	30	39	-3	1.25048	NA	
40	32	36	-2	1.43716	1.36014	-0.07702
40	44	37	-1.5	1.34287	1.48206	0.13919
27	30	NA	3		NA	
44	43	48	2		NA	
29	30	25	-2		NA	
24	9	NA	-15		NA	
41	43	38	-1.5	1.47044	1.46141	-0.00903
29	23	25	-2	1.3896	1.34894	-0.04066
48	45	NA	-3		NA	
29	12	5	-12	1.06972	1.02778	-0.04194
47	62	NA	15		NA	
34	39	31	-1.5		NA	
29	27	NA	-2		NA	
18	17	15	-1.5		NA	
54	54	53	-0.5		NA	
51	50	56	2.5	1.37212	1.1654	-0.20672
57	58	63	3	1.39338	1.37262	-0.02076
24	27	27	1.5	1.21602	1.23319	0.01717
50	33	43	-3.5		NA	
23	15	NA	-8		NA	
50	60	63	6.5	1.17221	1.12344	-0.04877
29	33	40	5.5	1.16093	1.21438	0.05345
50	46	51	0.5	1.28636	1.26478	-0.02158
35	28	25	-5		NA	
31	33	24	-3.5	1.23546	1.21227	-0.02319
39	26	42	1.5	1.15933	1.14146	-0.01787
17	14	23	3		NA	
37	40	41	2		NA	
38	40	41	1.5		NA	
24	20	22	-1	1.27013	1.30773	0.0376
44	29	30	-7		NA	
26	25	27	0.5	1.36122	1.19915	-0.16207
41	23	33	-4	1.08095	1.04409	-0.03686
38	41	45	3.5	1.16164	1.14756	-0.01408
40	28	38	-1	1.18596	1.17987	-0.00609
30	23	21	-4.5	0.965859	0.941869	-0.02399

56	55	54	-1	1.20305	1.19017	-0.01288
51	57	56	2.5		NA	
28	34	31	1.5		NA	
49	34	38	-5.5	1.29717	1.23022	-0.06695
20	24	18	-1	1.17764	1.16986	-0.00778
56	53	45	-5.5		NA	
34	30	38	2	1.16393	1.19377	0.02984
41	59	NA	18	1.33179	NA	
44	46	33	-5.5	1.15605	1.11559	-0.04046
31	23	32	0.5		NA	
19	15	NA	-4		NA	
26	28	30	2	1.04743	1.05911	0.01168
31	24	29	-1	1.17078	1.0736	-0.09718
34	21	23	-5.5		NA	
48	41	54	3	1.27031	1.23238	-0.03793
31	27	24	-3.5		NA	
39	42	46	3.5		NA	
30	32	29	-0.5		NA	
38	40	NA	2	1.36479	1.40814	0.04335
52	55	52	0		NA	
23	18	20	-1.5		NA	
36	32	30	-3		NA	
32	25	NA	-7		NA	
35	30	36	0.5		NA	
39	31	30	-4.5		NA	
28	28	35	3.5	1.1364	1.09075	-0.04565
46	42	38	-4	1.223	1.231	0.008
23	22	20	-2		NA	
31	36	39	4		NA	
57	49	55	-1		NA	
37	34	36	-0.5	1.25417	1.28035	0.02618
32	28	28	-2		NA	
45	36	36	-4.5		NA	
16	20	21	2.5		NA	
26	21	27	0.5	0.971423	0.978316	0.006893
44	47	48	2		NA	
29	34	25	-2		NA	
45	51	51	3		NA	
46	48	51	2.5		NA	
49	49	57	4	1.41345	1.42475	0.0113
51	42	36	-7.5	0.9756	1.01482	0.03922
29	23	18	-5.5	1.08067	1.00706	-0.07361
38	36	43	2.5	1.30839	1.20557	-0.10282

48	46	38	-5		NA	
31	29	30	-0.5	1.14216	1.1561	0.01394
23	23	15	-4	0.937324	0.990719	0.053395
55	53	58	1.5		NA	
39	35	36	-1.5		NA	
24	32	30	3		NA	
64	65	48	-8		NA	
50	NA	32	-9		NA	
62	60	65	1.5	1.20804	1.1396	-0.06844
41	32	30	-5.5	1.61123	1.59819	-0.01304
21	21	18	-1.5		NA	
26	30	27	0.5		NA	
39	33	42	1.5	1.22044	1.23063	0.01019
34	43	43	4.5	1.61968	1.63031	0.01063
46	37	39	-3.5	1.53017	1.49108	-0.03909
29	36	26	-1.5		NA	
45	NA	38	-3.5		NA	
38	44	36	-1		NA	
50	49	52	1	1.44154	1.47896	0.03742
30	31	25	-2.5		NA	
22	31	22	0	1.52211	1.20973	-0.31238
28	26	27	-0.5	1.06222	1.09278	0.03056
33	33	38	2.5		NA	
30	20	NA	-10		NA	
14	21	20	3		NA	
36	29	42	3	1.28147	NA	
39	43	46	3.5	1.13638	1.14037	0.00399
44	39	29	-7.5	1.13482	0.995948	-0.138872
51	36	42	-4.5		NA	
26	30	32	3	1.01948	0.973865	-0.045615
48	41	38	-5		NA	
31	19	NA	-12		NA	
36	43	42	3		NA	
18	23	21	1.5	1.12348	1.08178	-0.0417
51	51	37	-7		NA	
25	27	17	-4	1.09944	1.03879	-0.06065
19	10	9	-5	0.971031	0.943742	-0.027289
24	30	20	-2		NA	
42	42	40	-1	1.1408	1.10174	-0.03906
24	24	20	-2	1.31015	1.29976	-0.01039
31	30	31	0	1.1204	1.01965	-0.10075
41	34	48	3.5	1.27385	1.37121	0.09736
39	39	45	3		NA	

27	29	NA	2	1.20713	1.27004	0.06291
51	51	46	0	1.09659	1.17219	0.0756
43	42	45	1		NA	
26	28	25	-0.5	1.1661	1.17083	0.00473
31	31	27	-2		NA	
34	38	33	-0.5	1.04544	NA	
35	34	23	-6	1.1351	1.02383	-0.11127
25	19	22	-1.5		NA	
40	48	46	3		NA	
25	21	28	1.5	1.27073	1.38337	0.11264
32	30	29	-1.5	1.02659	0.984392	-0.042198
21	10	NA	-11		NA	
40	30	31	-4.5		NA	
28	25	21	-3.5		NA	
32	27	22	-5	1.11964	1.02415	-0.09549
20	21	22	1		NA	
15	10	NA	-5	0.98364	0.957876	-0.025764
32	25	22	-5	1.22637	1.14108	-0.08529
37	39	NA	2	1.13507	1.19326	0.05819
21	19	17	-2	1.07846	1.11461	0.03615
22	26	21	-0.5		NA	
56	38	NA	-18	1.40926	1.46149	0.05223
48	52	52	2		1.24474	
22	6	8	-7		NA	
27	36	32	2.5		NA	
22	24	24	1		NA	
51	40	53	1		NA	
26	23	25	-0.5	1.1083	1.06188	-0.04642
22	19	17	-2.5	1.15125	1.12485	-0.0264
17	24	20	1.5	0.965748	0.943594	-0.022154
49	43	49	0		NA	
20	20	18	-1		NA	
29	32	43	7	1.18747	1.1278	-0.05967
35	40	42	3.5	1.29438	1.21985	-0.07453
34	33	36	1	1.58438	1.58945	0.00507
30	28	NA	5		NA	
28	23	22	-3		NA	
34	30	NA	-4	0.974226	0.966671	-0.007555
41	52	54	6.5		NA	
23	28	21	-1	1.14215	1.09739	-0.04476
40	28	31	-4.5	1.06991	1.01855	-0.05136
24	25	24	0	0.972459	0.948903	-0.023556
27	24	16	-5.5		NA	

27	29	26	-0.5	1.36257	1.33043	-0.03214
38	32	NA	-6		NA	
21	24	NA	3	1.23251	1.09358	-0.13893
21	22	21	0		NA	
31	34	41	5		NA	
31	32	20	-5.5	1.1504	1.19244	0.04204
51	42	NA	-9	1.20965	1.17505	-0.0346
54	63	NA	9		NA	
29	30	33	2		NA	
63	51	60	-1.5		NA	
37	35	28	-4.5	1.44836	1.33509	-0.11327
32	31	35	1.5		NA	
37	40	37	0	1.24359	1.23024	-0.01335
39	45	41	1		NA	
28	29	28	0	1.1697	1.1502	-0.0195
19	21	23	2	1.2524	1.22042	-0.03198
43	43	37	-3	1.47994	1.50001	0.02007
45	48	47	1	1.38803	1.38192	-0.00611
24	29	NA	5	1.22087	1.24227	0.0214
39	38	37	-1	1.2783	1.20802	-0.07028
30	35	37	3.5	1.31704	1.34659	0.02955
44	51	55	5.5	1.29339	1.39874	0.10535
43	45	40	-1.5		NA	
49	50	56	3.5	1.36757	1.26856	-0.09901
35	33	23	-6	1.11913	1.09326	-0.02587
31	33	31	0	1.14889	1.18907	0.04018
36	33	37	0.5		NA	
45	45	47	1	1.16469	1.16816	0.00347
40	33	46	3	1.35312	1.34428	-0.00884
51	45	42	-4.5		NA	
44	29	34	-5		NA	
29	31	NA	2		NA	
24	28	28	2		NA	
39	40	37	-1		NA	
42	52	57	7.5		NA	
29	28	41	6		NA	

Hippocampal volume				CSF ptau		
Baseline	1 year	2 year	Annual char	Baseline	1 year	Annual char
6869	6451	NA	-418	13.29	13.3	0.01
7075	6832	6599	-238	33.43	34.04	0.61
6730	6693	6280	-225	16.68	NA	
7309	7331	7307	-1	15.88	19.88	4
8310	8300	8289	-10.5	16.74	17.47	0.73
8154	7961	NA	-193	13.45	NA	
7595	7323	7665	35	12.5	15.44	2.94
4087	3858	3471	-308	18.23	18.4	0.17
5268	NA	NA		58.65	61.47	2.82
6914	6898	NA	-16	21.4	21.4	0
6232	6363	6156	-38	28.4	25.79	-2.61
7606	7550	NA	-56	12.23	11.55	-0.68
6767	6048	NA	-719	30.25	28.04	-2.21
7389	7486	NA	97	16.69	14.66	-2.03
7262	7431	7181	-40.5	22.08	22.39	0.31
7766	7607	NA	-159	20.87	21.01	0.14
4764	4472	4082	-341	65.4	64.91	-0.49
7713	7723	7644	-34.5	14.88	15.52	0.64
7465	7091	7070	-197.5	12.17	11.78	-0.39
7608	7694	7438	-85	18.06	17.64	-0.42
6527	6350	6018	-254.5	14.62	14.19	-0.43
7510	NA	7230	-140	16.31	NA	
5572	5266	NA	-306	38.64	38.15	-0.49
8255	8049	8003	-126	24.43	26.55	2.12
7859	7889	7217	-321	14.38	NA	
5792	6176	5550	-121	26.3	26.48	0.18
4946	4373	4077	-434.5	24.94	26.06	1.12
5832	5734	5938	53	47.6	46.98	-0.62
6951	6628	6660	-145.5	29.3	30.94	1.64
5881	5661	5712	-84.5	40.9	47.14	6.24
5381	5137	5419	19	21.41	NA	
7774	7913	7677	-48.5	16.77	15.6	-1.17
5675	5516	5432	-121.5	20.29	20.66	0.37
7691	7597	7571	-60	18.54	20.41	1.87
7382	6943	6911	-235.5	48.14	48.81	0.67
5565	5118	5086	-239.5	41.03	30.86	-10.17
7751	7659	NA	-92	19.67	19.22	-0.45
4645	5084	4703	29	22.91	23.92	1.01
5372	NA	4822	-275	27.12	27.53	0.41

5951	6318	6039	44	32.32	31.17	-1.15
6221	6222	5960	-130.5	15.47	15.34	-0.13
5893	NA	5321	-286	32.75	28.47	-4.28
7745	7437	7273	-236	37.99	43.14	5.15
5495	5252	5277	-109	13.1	11.97	-1.13
7068	6894	6839	-114.5	34.73	39.29	4.56
7164	7015	NA	-149	41.39	39.2	-2.19
7639	NA	NA		15.29	NA	
8209	NA	8003	-103	16.71	16.33	-0.38
5561	5264	5292	-134.5	39.86	37.75	-2.11
5622	5011	NA	-611	45.17	45.07	-0.1
4868	4614	4290	-289	29.26	33.3	4.04
5151	NA	NA		78.46	84.4	5.94
7036	7157	6765	-135.5	13.21	12.45	-0.76
7817	NA	7381	-218	16.77	15.68	-1.09
7225	6714	6388	-418.5	31.59	32.95	1.36
7634	NA	7467	-83.5	14.57	NA	
7509	7383	6826	-341.5	36.23	39.69	3.46
9104	9183	NA	79	25.42	25.79	0.37
6905	6995	6719	-93	10.57	10.6	0.03
8640	8615	8654	7	15.55	16.85	1.3
7203	6980	6886	-158.5	24.9	28.5	3.6
4737	4704	NA	-33	20.37	NA	
5887	6126	6042	77.5	20.87	18.54	-2.33
5839	NA	5159	-340	37.96	NA	
6931	6903	NA	-28	14.12	NA	
6257	5788	5881	-376	23.98	22.19	-1.79
6140	NA	NA		36.47	34.89	-1.58
9433	NA	9054	-189.5	14.25	NA	
7880	7489	7478	-402	22.8	26.18	3.38
6102	5947	NA	-155	35.95	36.99	1.04
6410	NA	NA		34.06	34.4	0.34
5388	5100	NA	-288	15.57	NA	
5904	5677	5576	-164	31.48	NA	
6289	6095	5739	-275	35.77	33.23	-2.54
6498	6576	6550	26	10.41	11.26	0.85
6535	6083	6039	-248	29.13	NA	
7749	7889	7763	7	15.07	15.41	0.34
6443	6103	NA	-340	18.34	17.64	-0.7
8764	8683	8479	-142.5	12.95	12.92	-0.03
4445	4421	4353	-46	12.46	11.63	-0.83
5527	5248	5010	-258.5	39.15	27.22	-11.93
8609	NA	NA		20.13	21.82	1.69

7651	NA	7327	-162	15.82	14.99	-0.83
4425	4493	NA	68	29.77	30.85	1.08
4692	4582	4588	-52	20.69	21.71	1.02
7360	7494	7102	-129	19.43	NA	
8149	7900	8124	-12.5	16.98	19.4	2.42
6490	6434	6124	-183	30.47	30.98	0.51
8379	NA	NA		23.7	25.23	1.53
6452	NA	6209	-121.5	39.89	37.5	-2.39
7665	8144	8534	434.5	18.66	17.28	-1.38
7859	NA	7444	-207.5	42.85	47.01	4.16
4361	4234	3993	-184	23.74	22.08	-1.66
4913	4763	4585	-164	42.03	42.21	0.18
7134	NA	6609	-262.5	15.06	15.59	0.53
8489	8406	8366	-61.5	13.56	14.75	1.19
7011	6722	6823	-94	40.41	42.04	1.63
4984	5039	4972	-6	21.53	23.19	1.66
7336	6808	NA	-528	24.16	NA	
6107	5580	NA	-527	31.69	29.71	-1.98
8012	8105	8003	-4.5	27.56	30.97	3.41
5896	5756	NA	-140	9.75	10.97	1.22
7831	7323	7748	-41.5	28.19	24.25	-3.94
6284	NA	5589	-347.5	22.19	22.62	0.43
7862	8155	NA	293	25.34	NA	
5825	NA	NA		59.86	56.07	-3.79
5561	5205	5210	-175.5	44.42	45.12	0.7
7446	7455	7365	-40.5	11.86	10.63	-1.23
6665	6623	6737	36	16.13	NA	
6138	NA	NA		37.03	NA	
6675	6677	6627	-24	17.58	17.36	-0.22
6796	6502	5914	-441	32.63	34.78	2.15
7534	7440	7157	-188.5	14.64	15.93	1.29
4581	4279	NA	-302	24.87	NA	
8484	8709	NA	225	20.83	22.11	1.28
5422	NA	4617	-402.5	21.22	23.47	2.25
5855	NA	NA		21.4	21.35	-0.05
7161	7056	6625	-268	22.57	24.64	2.07
5660	5542	4957	-351.5	28.06	26.32	-1.74
5511	NA	NA		34.65	34.66	0.01
7619	7251	NA	-368	46.32	NA	
6250	5833	5754	-248	37.9	38.02	0.12
6979	6635	NA	-344	51.42	NA	
7549	NA	NA		12.57	NA	
7202	7044	6875	-163.5	21.39	19.75	-1.64

	5978	5727	NA	-251	28.6	NA	
	6748	6299	6239	-254.5	28.29	29.78	1.49
	8581	8168	NA	-413	9.35	NA	
	7739	NA	7431	-154	27.33	26.12	-1.21
	4600	NA	NA		22.82	22.47	-0.35
	7142	NA	NA		17.77	16.58	-1.19
	6124	6017	5733	-195.5	23.8	23.67	-0.13
	5702	5775	5617	-42.5	31.94	37.99	6.05
	7409	NA	NA		16.84	17.12	0.28
	7196	7069	7228	16	16.07	17.07	1
	6135	5699	5878	-128.5	26.61	23.65	-2.96
	6180	6136	NA	-44	62.48	72.41	9.93
	5995	5681	3737	-1129	20.8	20.56	-0.24
	5721	5316	5025	-348	32.46	31.23	-1.23
	6112	5795	5481	-315.5	34.36	NA	
	6048	NA	NA		13.99	14.74	0.75
	3281	NA	NA		17.01	17.95	0.94
	7498	7265	6954	-272	22.48	21.94	-0.54
	5296	5171	NA	-125	39.84	44.19	4.35
NA		5236	NA		34.93	35.47	0.54
NA		NA	NA		18.07	19.75	1.68
NA		NA	NA		13.39	NA	
NA		6398	6141	-257	15.97	16.71	0.74
NA		5528	NA		27.85	25.87	-1.98
NA		5422	NA		16.23	16.9	0.67
NA		4638	NA		32.01	34.38	2.37
NA		6658	NA		16.32	NA	
NA		NA	NA		31.9	32.01	0.11
NA		6876	NA		45.23	48.41	3.18
NA		5968	5976	8	33.87	35.11	1.24
NA		6927	6783	-144	21.45	22.35	0.9
NA		NA	5120		40.69	39.09	-1.6
NA		NA	NA		14.99	14.77	-0.22
NA		NA	NA		25.18	NA	
NA		7222	7005	-217	20.65	21.56	0.91
NA		NA	NA		58.05	36.59	-21.46
NA		6311	5890	-421	37.97	NA	
NA		4478	NA		15.43	NA	
NA		6746	6766	20	34.21	37.88	3.67
NA		5671	5741	70	69.63	67.04	-2.59
NA		NA	NA		23.71	21.65	-2.06
NA		7496	NA		19.86	17.39	-2.47
NA		5146	4808	-338	37.44	30.98	-6.46

NA	6004	5980	-24	26.56	26.35	-0.21
NA	3948	NA		22.9	NA	
NA	NA	NA		37.45	35.86	-1.59
NA	7106	NA		25.96	NA	
NA	NA	NA		21.45	20.12	-1.33
NA	4152	NA		25.27	23.46	-1.81
6080	NA	NA		37.51	34.28	-3.23
6647	6356	NA	-291	18.05	17.13	-0.92
6410	6495	5909	-250.5	21.85	24.89	3.04
7206	NA	6636	-285	22.55	26.1	3.55
7821	7724	NA	-97	33.02	NA	
7113	7066	6957	-78	30.66	30.06	-0.6
NA	6030	NA		21.69	NA	
7848	7708	7769	-39.5	21.19	21.91	0.72
5960	NA	NA		24.75	19.68	-5.07
NA	NA	NA		24.04	24.36	0.32
8446	8147	8059	-193.5	21.84	21.75	-0.09
7677	7846	7643	-17	27.73	28.51	0.78
NA	NA	NA		40.96	NA	
6787	6902	6915	64	31.71	29.59	-2.12
7383	7494	7331	-26	27.12	NA	
7655	7412	7684	14.5	19.71	23.83	4.12
7363	7535	7135	-114	29.6	NA	
6079	6088	NA		32.15	29.93	-2.22
6025	6164	5642	-191.5	34.68	34.98	0.3
6530	6435	6504	-13	19.69	20.04	0.35
7194	6981	7125	-34.5	22.82	23.96	1.14
7467	7051	6771	-348	22.1	20.97	-1.13
7665	7698	7701	18	18.7	19.7	1
7438	7464	NA	26	17.65	NA	
7076	6716	6685	-195.5	32.75	31.56	-1.19
6729	6731	NA	2	25.56	NA	
6069	NA	5375	-347	19.71	19.13	-0.58
7458	7149	7250	-104	18.66	NA	
6986	7056	7171	92.5	8	NA	
6173	NA	6177	2	8	8	0

	**CSF Aβ**	
Baseline	1 year	Annual change
1501	1176	-325
547.3	472.8	-74.5
1582		
550.6	682.7	132.1
1647	1466	-181
874.1		
1526	1700	174
1258	766.5	-491.5
383.3	373.3	-10
1410	1257	-153
352.5	326.1	-26.4
1048	988.7	-59.3
597.1	388.6	-208.5
1605	1410	-195
1288	929.8	-358.2
1427	966.9	-460.1
792.7	668.6	-124.1
1414	1306	-108
915.3	682	-233.3
776.6	711.4	-65.2
453.2	411.8	-41.4
1652		
580.2	498.5	-81.7
498.6	464.4	-34.2
1226		
1566	1553	-13
536.8	676.8	140
817	756.6	-60.4
991.3	1042	50.7
670.7	699.6	28.9
1367		
942.8	911	-31.8
282.9	273.9	-9
1385	1442	57
683.1	731.9	48.8
751.5	609.6	-141.9
1028	1050	22
1338	1303	-35
288.4	279.2	-9.2

803.6	639.9	-163.7
1520	1614	94
576.8	500.1	-76.7
844.4	867.1	22.7
941.7	809.8	-131.9
888.1	858.3	-29.8
838.6	777.4	-61.2
1391		
1405	1650	245
588	585.3	-2.7
854.2	624.8	-229.4
556.7	547	-9.7
414.1	371.4	-42.7
1148	1344	196
1168	1291	123
332.6	313.6	-19
1100		
333.5	336.2	2.7
1327	963.6	-363.4
1006	934	-72
1014	1139	125
831.3	878	46.7
1634		
1645	1447	-198
606		
1203		
500.5	344	-156.5
477.6	448.6	-29
1495		
649.7	557	-92.7
594	514.1	-79.9
568.6	611.5	42.9
1600		
449.8		
627.5	639.2	11.7
826.8	873	46.2
540.2		
1263	1443	180
821.4	618.1	-203.3
806.8	865.4	58.6
461.6	467.9	6.3
648.4	548.7	-99.7
560.6	416.6	-144

1245	1118	-127
682.5	642.1	-40.4
368.6	320.7	-47.9
1429		
1251	1381	130
1079	1114	35
1117	1143	26
507.3	499.7	-7.6
1323	950	-373
467	472	5
269.3	212.3	-57
669.3	585.8	-83.5
1697	1608	-89
657.6	958.6	301
803.5	833.1	29.6
566.7	648.8	82.1
493.4		
957.8	785.4	-172.4
744.2	615.7	-128.5
594.2	544.1	-50.1
742	676.3	-65.7
1038	620.1	-417.9
536.8		
1081	988.9	-92.1
730.1	668.7	-61.4
1298	994.1	-303.9
1271		
421.3		
1208	997.4	-210.6
908.9	760.6	-148.3
1314	863.4	-450.6
509.7		
1396	1664	268
293.7	301	7.3
1435	1098	-337
579.2	482.5	-96.7
755.1	481.8	-273.3
603.1	448.8	-154.3
696		
657.6	653.7	-3.9
699.3		
1449		
561.3	423.7	-137.6

907.3		
685.5	632.6	-52.9
670.5		
1516	1603	87
566.3	580.6	14.3
1330	1465	135
912	796.4	-115.6
649.1	674.1	25
1643	1488	-155
1470	1056	-414
860.9	664.6	-196.3
596.6	592.5	-4.1
291.1	273.5	-17.6
570	589.7	19.7
761.5		
1113	1053	-60
689.2	698.5	9.3
390.5	400.5	10
521.2	614.3	93.1
673	643.2	-29.8
414.6	437.1	22.5
805.2		
1477	1343	-134
539.3	482.2	-57.1
1467	1297	-170
674.5	827.2	152.7
540.6		
620.3	535.4	-84.9
702.1	618.1	-84
643.4	674.8	31.4
1614	1618	4
529.2	503.8	-25.4
1379	1039	-340
1479		
668.2	557.3	-110.9
638.3	595.2	-43.1
526.4		
1243		
935.3	1044	108.7
657.9	638.3	-19.6
619.8	660.2	40.4
1542	1285	-257
574	469.6	-104.4

496.4	418.5	-77.9
1275		
614.4	480.4	-134
907		
1685	1700	15
857.2	722.1	-135.1
1700	1700	0
1700	1700	0
1700	1700	0
1700	1700	0
1700		
1700	1700	0
1700		
1700	1700	0
1700	1657	-43
1700	1443	-257
1700	1700	0
1700	1700	0
1700		
1700	1627	-73
1700		
1700	1700	0
1700		
1700	1700	0
1700	1689	-11
1700	1527	-173
1700	1700	0
1700	1540	-160
1700	1700	0
1700		
1700	1700	0
1700		
1700	1452	-248
1700		
390.9		
428.8	419.6	0
